# The Role of the Lowest Excited Triplet State in Defining the Rate of Photoaquation of Hexacyanometalates

**DOI:** 10.1021/acs.jpclett.3c02775

**Published:** 2024-01-02

**Authors:** Eric J. Mascarenhas, Mattis Fondell, Robby Büchner, Sebastian Eckert, Vinicius Vaz da Cruz, Alexander Föhlisch

**Affiliations:** † Institute for Methods and Instrumentation for Synchrotron Radiation Research, Helmholtz-Zentrum Berlin für Materialien und Energie GmbH, 12489 Berlin, Germany; ‡ Institute of Physics and Astronomy, Universität Potsdam, 14476 Potsdam, Germany

## Abstract

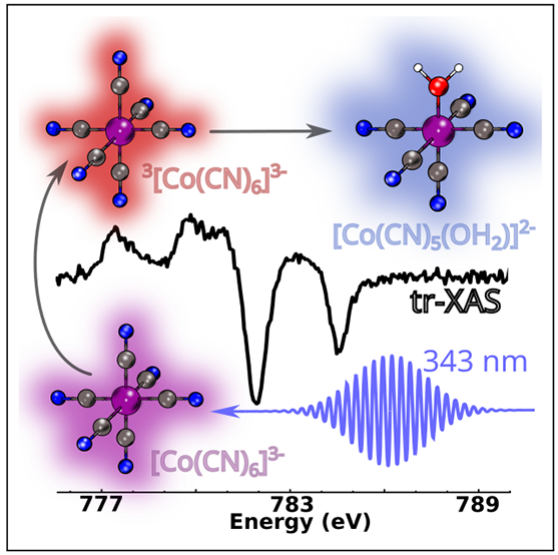

Photosolvation is a type of ligand substitution reaction started by irradiation of a solution with light, triggering the replacement of a ligand with a molecule from the solvent. The excited state is created through many possible pathways. For the class of hexacyanides of groups 8 and 9 of the periodic table, irradiation in the ligand field band is followed by intersystem crossing to the lowest excited triplet state, which we propose to mediate the photoaquation reaction in this class of complexes. In this study, we present time-resolved X-ray absorption data showing indications of the triplet intermediate state in the cobalt(III) hexacyanide complex and we discuss general aspects of the photoaquation reaction in comparison with reported data on the isoelectronic iron(II) hexacyanide. Quantum chemical calculations are analyzed and suggest that the nature of the lowest excited triplet state in each complex can explain the drastically different rate of reactions observed.

Ligand substitution reactions constitute a major class of processes in inorganic chemistry that involve the replacement of an existing ligand within a metal complex by a new one. These are important reaction steps toward synthesis of systems with a desired functionality. However, not all sought-after substitutions take place in the ground-state potential energy surface, often necessitating the use of light to drive a certain reaction. In this context, the most fundamental type of photoinduced ligand substitution is photosolvation. These are reactions in which a ligand is ejected from a complex and is thereafter replaced by a molecule from the solvent. Photosolvation reactions are crucial building blocks for rationalizing more complex steps in reaction mechanisms as well as for investigating complex–solvent interactions and the role of the solvent on a complex’s properties.

In this context, metal hexacyanides, as a result of their high stability and symmetry, constitute an excellent testing ground of the systematic behavior of metal complexes in terms of their basic geometrical, vibrational, and electronic structure.^1−4^ Within this class, [Fe(CN)_6_]^4–^ represents one of the most studied cases.^5−13^ Its low-spin d^6^ configuration, accompanied by the high covalency, gives this compound the necessary parameters for great control of properties. Also with an equivalent d^6^ configuration, the cobalt(III) hexacyanometalate exhibits a very similar electronic structure and properties. Despite great insight from the ferrocyanide system, the isoelectronic cobalt counterpart, [Co(CN)_6_]^3–^ has remained underexplored since the beginning of interest in hexacyanometalate systems.

Both d^6^ hexacyanides, when excited to their ligand field (LF) transition, undergo photosolvation through a dissociative process.^14^ In [Fe(CN)_6_]^4–^, the created ^1^T_1g_ excited state falls into a dissociative ^3^T_1g_ state through intersystem crossing^12^ with the photo aquated species being formed in a maximum quantum yield of 0.89.^5,15^ In spite of considerable recent efforts, several questions remain about the exact mechanism of this photoreaction. The same mechanism was proposed for [Co(CN)_6_]^3–^^16^ taking into account the reactivity of the triplet state (see Figure 1); however, despite all similarities, the photoaquation reaction, following excitation to the LF state, is observed with a much lower quantum yield of 0.31^17^ for this system.

**Figure 1:**
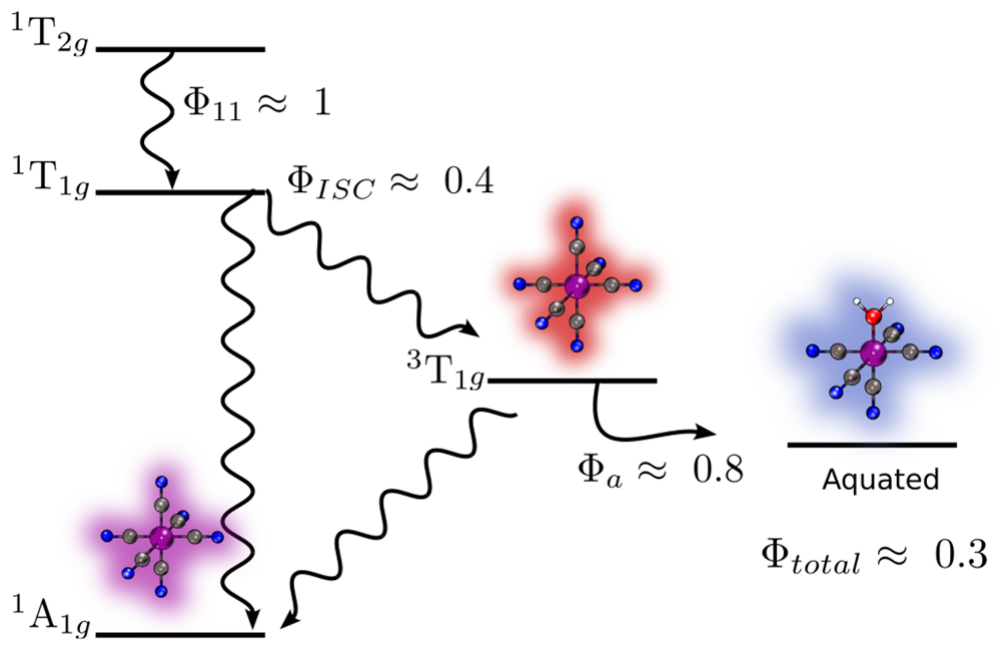
Diagram displaying the steps involved from photoexcitation to photoaquation of [Co(CN)_6_]^3–^. Φ stands for the quantum yield of each process indicated by its index. The values were taken from the study of Scandola and Scandola.^16^

It puzzled many^1,2,18,19^ that the photoaquation of [Co(CN)_6_]^3–^ would not be as efficient as that of [Fe(CN)_6_]^4–^. Moreover, the cobalt complex does not suffer photo-oxidation but takes part in photoaquation in a wide range of wavelengths,^17^ increasing the confusing contrast between the two species.^5^ It was Scandola who performed photosensitization studies that elucidated the most probable mechanism (Figure 1) with the triplet state as the intermediate as proposed for the iron case.^16^ The short-lived nature of this transition state remained the greatest challenge for its detection and spectral characterization in [Fe(CN)_6_]^4–^. Although studies were performed for the [Co(CN)_6_]^3–^ in frozen matrices at very low temperatures,^20^ a characterization in a more realistic environment and in solution media, in which reactions are usually carried out, is preferable. Thus far, an explanation for the lower photoaqueous yields in [Co(CN)_6_]^3–^ has been lacking.

In recent decades, the development of synchrotron light sources with liquid microjets^21−24^ has allowed the analysis of chemical solutions in the soft X-ray domain.^25,26^ Especially, in time-resolved studies of transition metal complexes, distinct spectroscopic signatures at the metal L- and the ligand K-edges have enabled in-depth analysis of excited states as short-lived intermediates in photochemical cascades. The transitions at the metal L-edge enable one to probe the unoccupied levels centered at the metal, reporting on the occupation of the 3d orbitals. Meanwhile, the ligand edges can complementarily report on the unoccupied levels centered on the periphery of the complex. Such site-selectivity allows one to systematically dissect the bonding channels of the complex, yielding unique insight into the studies of chemical reactions and photochemistry^24,27−29^ This methodology is thus ideally suited to explore the properties of the electronic states involved in photoinduced ligand substitution reactions of cyanometalates and address the open questions regarding the reaction mechanism for different metal centers.

This study presents time-resolved X-ray absorption spectroscopy at the metal Co L_3,2_-edge and ligand N K-edge of [Co(CN)_6_]^3–^ after LF photoexcitation. Steady-state and time-resolved data will be presented for the complex in aqueous solution, and the results will be discussed and compared to *ab initio* quantum chemical computations. The results are combined to provide an explanatory picture for the crucial differences in the photoaquation yield between the isoelectronic [Co(CN)_6_]^3–^ and [Fe(CN)_6_]^4–^, focusing on the reactivity of the lowest triplet state.

Experiments were performed at the UE52-SGM^30^ beamline of BESSY II with the nm-Transmission NEXAFS end station.^22^ In every measurement, a concentration of 200 mM of K_3_[Co(CN)_6_] in deionized water was used. The system was excited with the third harmonic (343 nm) of a fiber laser system with a 1030 nm fundamental wavelength. All computations were performed with the ORCA^31^ package at the DFT/B3LYP^32^ level of theory with conductor-like polarizable continuum model^33^ and D3^34^ model with Becke–Johnson damping.^35^ Thereby the effects of solvation and dispersion were taken into account. Spectral computations were carried out using TD-DFT.^36^ Further details about the experiment and computations can be found in the Supporting Information.

As typical for the class of octahedral homoleptic d^6^ cyanide complexes,^3,13^ the electronic structure of [Co(CN)_6_]^3–^ is well understood by the interactions of the ligand-field split metal-d orbitals with σ and π ligand orbitals. The HOMO can be identified as the t_2g_ set of d orbitals of cobalt with an admixture from cyanide π orbitals. The LUMO, on the other hand, has strong contributions from the e_*g*_ set of the 3d shell, mixed with cyanide σ orbitals. The energy levels follow the same order as seen in the isoelectronic [Fe(CN)_6_]^4–^.^13^

The X-ray absorption spectra (XAS) at the N K- and Co L_3,2_-edges are presented in Figure 2. At the N K-edge (Figure 2a), the XAS of [Co(CN)_6_]^3–^ presents a single broad peak centered at 399.5 eV with a shoulder centered at 398.6 eV. At the metal L_3_-edge (Figure 2b), a peak is observed centered at 782.1 eV and is followed by a slightly smaller peak centered at 784.7 eV. The L_2_-edge has a lower intensity and spans from 794 to 802 eV centering at 796.6 and 799.8 eV. The steady-state measurements of the ligand K- and metal L_3_-edges agree well with the work reported by Lalithambika et al.^37^ The computed spectra at the TD-DFT level are plotted along with experimental data for each corresponding edge in Figure 2.

**Figure 2:**
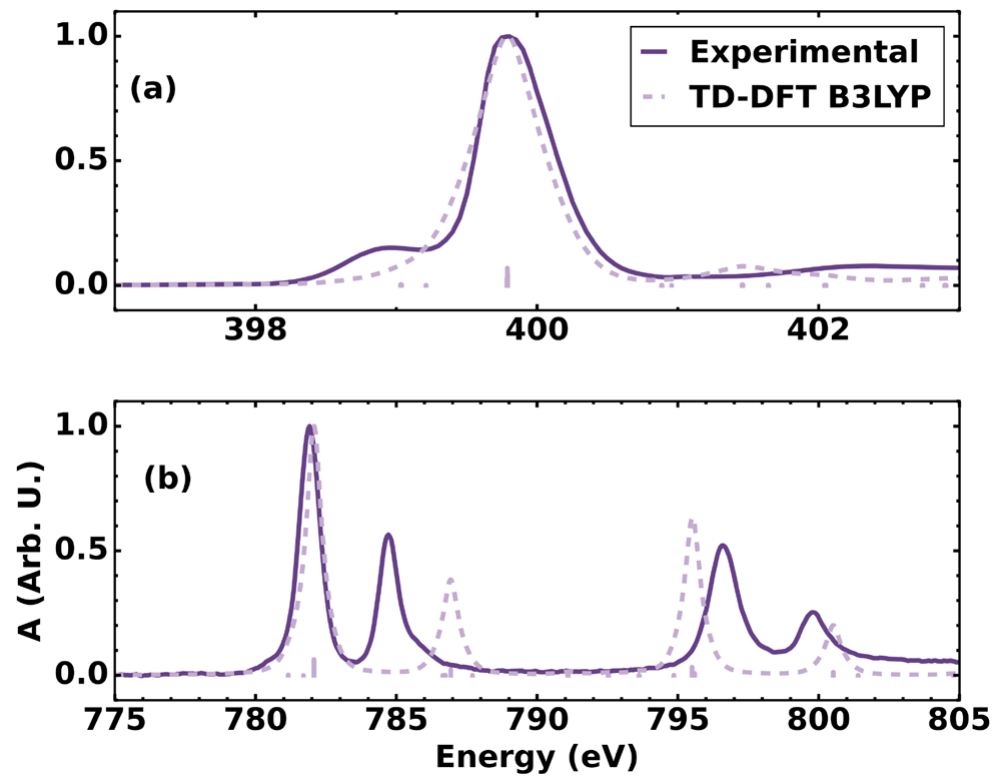
(a) Steady-state spectrum of an aqueous solution of K_3_[Co(CN)_6_] measured around the N K-edge compared to TD-DFT calculations. (b) Steady-state spectrum of an aqueous solution of K_3_[Co(CN)_6_] measured around the Co L_3,2_-edge compared with TD-DFT calculations, including spin–orbit coupling.

In the static spectrum at the N K-edge (Figure 2a), a strong signal centered at 399.5 eV is observed. This peak arises because of an electronic transition from the N 1s orbitals to the π set of virtual orbitals. As observed in Figure 2a, the shoulder seen in the experimental spectra is not well represented by the TD-DFT theory with respect to the distance to the main absorption peak. From the theoretical spectrum, however, it is possible to infer that this peak is due to weak contributions of transitions to the e_*g*_ set of orbitals with large cyanide σ amplitude.

The metal L_3_-edge, on the other hand, shows a pair of peaks as a result of electronic transitions from the 2p orbitals of the metal center to the virtual orbitals. The first strong peak seen is attributed to an electronic transition to the LUMO set of orbitals with e_*g*_ symmetry. The second peak, slightly weaker than the first, is attributed to transitions to a set of orbitals with π character mainly centered on the ligands and with a strong contribution from the t_2g_ set of d orbitals in the cobalt center. As seen through the TD-DFT computation and discussed by past studies,^6,13,38^ this transition intensity is proportional to the mixing of metal d orbitals and ligand π orbitals. The energy position, as also noticed in former studies on metal cyanides,^39−41^ is not well reproduced at the level of TD-DFT and even higher level theories. Qualitatively, however, and with the low computational cost of TD-DFT, the spectral shape is very well reproduced, and the intensity of the peak compared with the lower energy peak, a feature characteristic of this class of complexes, is also observed in the computation. The relative intensity of this peak, when compared with the main e_*g*_ peak at lower energy, is a measure of participation of the metal in back-bonding. In contrast to its iron counterpart, [Co(CN)_6_]^3–^ presents a lower degree of back-bonding as also noted by Lalithambika et al.^37^

Having established the static spectroscopic signatures, let us turn our attention to time-resolved measurements. In Figure 3, the time-resolved data for the system at the N K-edge is presented. The transient spectrum in Figure 3a was acquired at 100 ps after photoexcitation. A small but discernible 1 eV broad feature can be spotted centered at 396.0 eV and is highlighted in Figure 3d. A small absorption depletion centered at 398.2 eV is followed by a bleach centered at 398.8 eV and another absorption increase centered at 399.5 eV. Finally, another absorption depletion centered at 399.8 eV is observed. Delay traces were measured for two of the features, and the results are presented in Figure 3b. Computed spectra were considered for the assignment of the transient features at the N K-edge, and the analysis is presented in Figure 3c with details in Figure 3d for small but relevant features.

**Figure 3:**
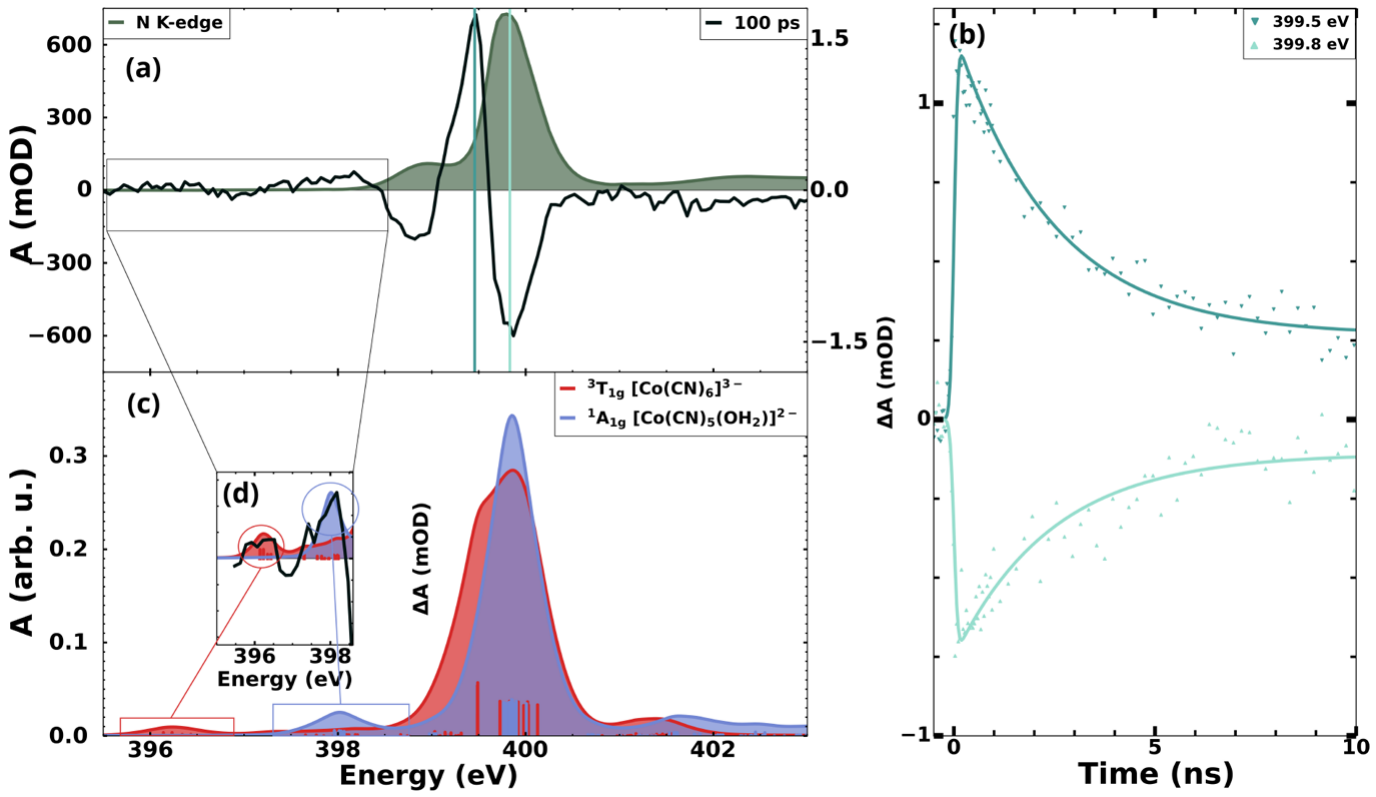
Time resolved X-ray absorption spectrum of aqueous K_3_Co(CN)_6_ at the N K-edge. (a) Transient spectrum acquired with a delay of 100 ps plotted along with the steady-state spectrum. (b) Delay traces measured at the main features. The lines in panel a indicate the energy position of the delay traces. (c) TD-DFT spectral calculations for the species considered to take part in the reaction. (d) A zoomed-in view of the computation showing the most discernible feature of the ^3^T_1g_ state of [Co(CN)_6_]^3–^ and of the ^1^A_1g_ state of the expected photoproduct [Co(CN)_5_(OH_2_)]^2–^ plotted along with the binned transient data.

As mentioned, the TD-DFT level of theory did not reproduce well the separation between LUMO and the ligand π manifold. Therefore, the experimental depletion observed in 398.6 eV is not theoretically represented. The main rise centered at 399.5 eV has contributions from both the aquated photoproduct and the intermediary triplet state due to the broadening expected with the lowering of symmetry. The observed bleaching centered on the same energy as the main peak in the static spectrum is mainly attributed to the depletion of starting [Co(CN)_6_]^3–^, which has the strongest absorption at this photon energy.

The computed spectra shown in Figure 3c show that all three species considered have overlapping signatures at their most intense absorption band around 400 eV. However, zooming in on the area below 399 eV (see Figure 3d), it is possible to distinguish some features of the considered intermediate, the ^3^T_1g_ state of [Co(CN)_6_]^3–^, and the final aquated product, [Co(CN)_5_(OH_2_)]^2–^. The computation shows a weak feature centered at 398.0 eV, while the triplet species presents an isolated and slightly smaller peak centered at 396.2 eV. Strikingly, the transient spectrum shown in Figure 3a shows some weak features that agree with the proposed structures. A binned version of the data plotted with the computations and zoomed-in for clarity is shown in Figure 3d. The highlighted region is provided without binning in the Supporting Information. These proposed assignments are strengthened by careful analysis of the data at the Co L_3_-edge.

Time-resolved data at the Co L_3_-edge are presented in Figure 4. The transient spectrum in Figure 4a was acquired 100 ps after photoexcitation. The ground-state absorption spectrum is plotted for comparison. The spectrum exhibits a prominent increase in absorption centered at 777.6 eV followed by another rise of similar intensity centered at 780.0 eV. These features are clear indications of the depopulation of the t_2g_ orbitals. A small shoulder centered at 781.1 eV precedes the main ground-state bleach, centered at 782.1 eV. Lastly, a second ground-state bleaching is observed in the scanned range and is centered at 784.6 eV. The delay traces are shown in Figure 4c and were measured at the maximum of each feature highlighted as well as at the main ground-state depletion.

**Figure 4:**
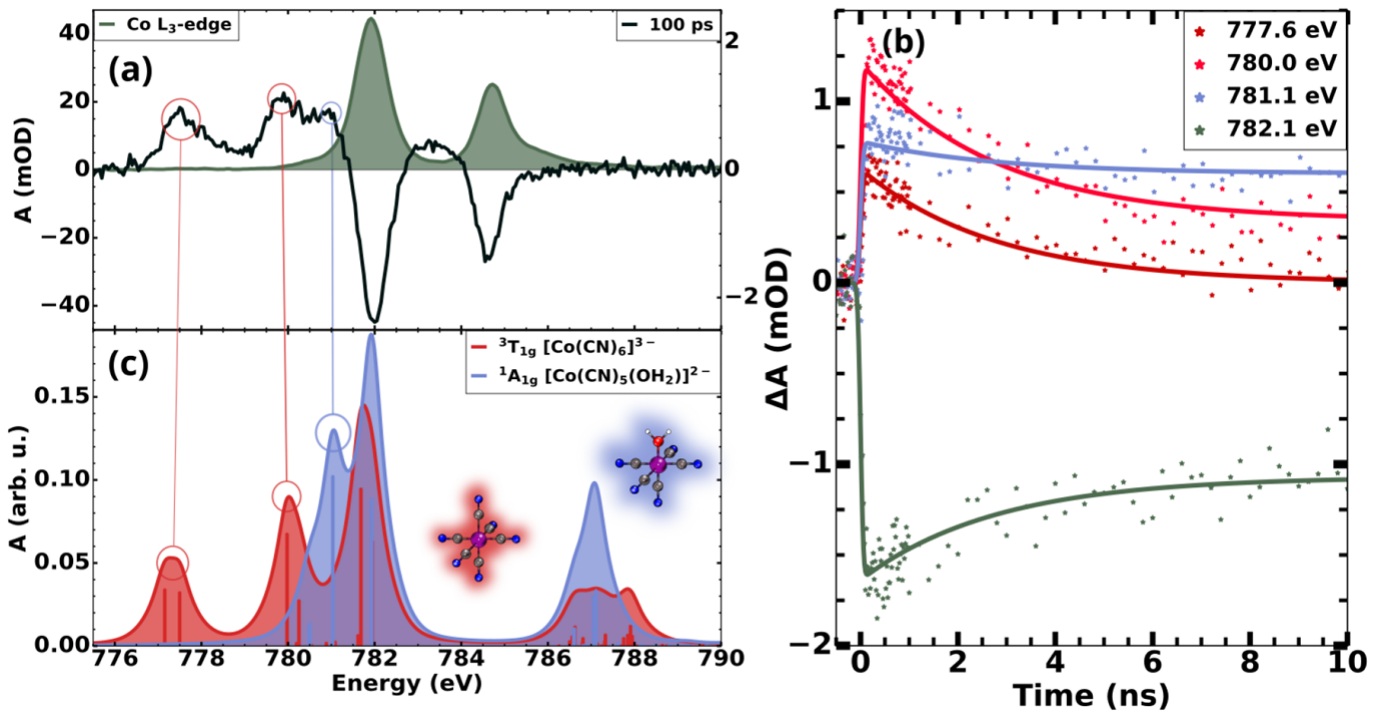
Transient spectra of aqueous K_3_Co(CN)_6_ at the Co L_3_-edge evidencing the long-lived triplet state and the photoaquated product. (a) Transient spectrum acquired with a delay of 100 ps plotted along with the steady-state spectrum. (b) Delay traces measured at the main features. The color of the data and fit matches the color of the plotted theoretical data for the features attributed to the ^3^T_1g_ state of [Co(CN)_6_]^3–^ and the ^1^A_1g_ state of the expected photoproduct, [Co(CN)_5_(OH_2_)]^2–^. The green curve is attributed mainly to ground-state depletion. (c) TD-DFT spectral calculations for the species considered to take part in the reaction.

The computed spectrum for the ^3^T_1g_ state of [Co(CN)_6_]^3–^ (Figure 4c) exhibits two peaks that coincide with the first two features observed in the experimental transient spectrum. The calculations presented in Figure 4c show that these bands are centered at 777.6 and 780.0 eV. From the computation, the lowest-energy peak is attributed to electronic transitions from the 2p orbitals of the cobalt center to the t_2g_ set of orbitals into the hole resulting from the LF excitation. The second peak, slightly higher than the first, is attributed to transitions to the lower-lying e_*g*_ orbital, which has the unpaired electron. The position and relative intensity of these peaks agree well with the first two features seen in the transient spectrum of Figure 4a and represent unambiguous signatures of the triplet state species. Interestingly, a clearly distinguishable feature associated with the photoaquated species is detected just below the main ground-state bleach, related to mixing between the e_*g*_ orbital with the orbitals of the water molecule.^13^Figure 4c shows that the TD-DFT calculation predicts a clear distinction from the triplet state at this region centered at 780.0 eV.

Looking at the delay traces presented in Figure 4b, we see strong indications that these features can be attributed to the considered species. Due to the higher contribution of the long-lived product in the absorption at 781.1 eV, this peak displays a different time constant, as seen in Figure 4b, and eventually asymptotically reaches a constant value. The global fit of the delay traces show that the first two peaks are associated with the short-lived decay component, showing a lifetime of 2.8 ns, which agrees well with data from Conti et al. for the lifetime of the ^3^T_1g_ state of [Co(CN)_6_]^3–^.^19^ On the other hand, the delay trace measured at 781.1 eV has a strong contribution from the long-lived component with a rise constant of 5.6 ns. The distinct behavior of the time traces enables a clear identification of both the ^3^T_1g_ state and the aquated photoproduct. Further details of the kinetic model and global fit are presented in the Supporting Information. The measured delay scan at 781.1 eV shows interesting agreement with the data acquired at the O K-edge, as will be discussed next.

The transient absorption changes at the K-edge are presented in Figure 5. The transient spectrum shown in Figure 5a was measured at a time delay of 5 ns. A single increase in absorption is observed, forming a broad peak centered at 532.4 eV. To rule out any processes being driven by the laser fluence, the experiment was repeated with the pure solvent, and no signal was observed. The transient measurement in the pure solvent can be found in the Supporting Information. The pump–probe delay-dependent intensity of this feature is presented in Figure 5b.

**Figure 5:**
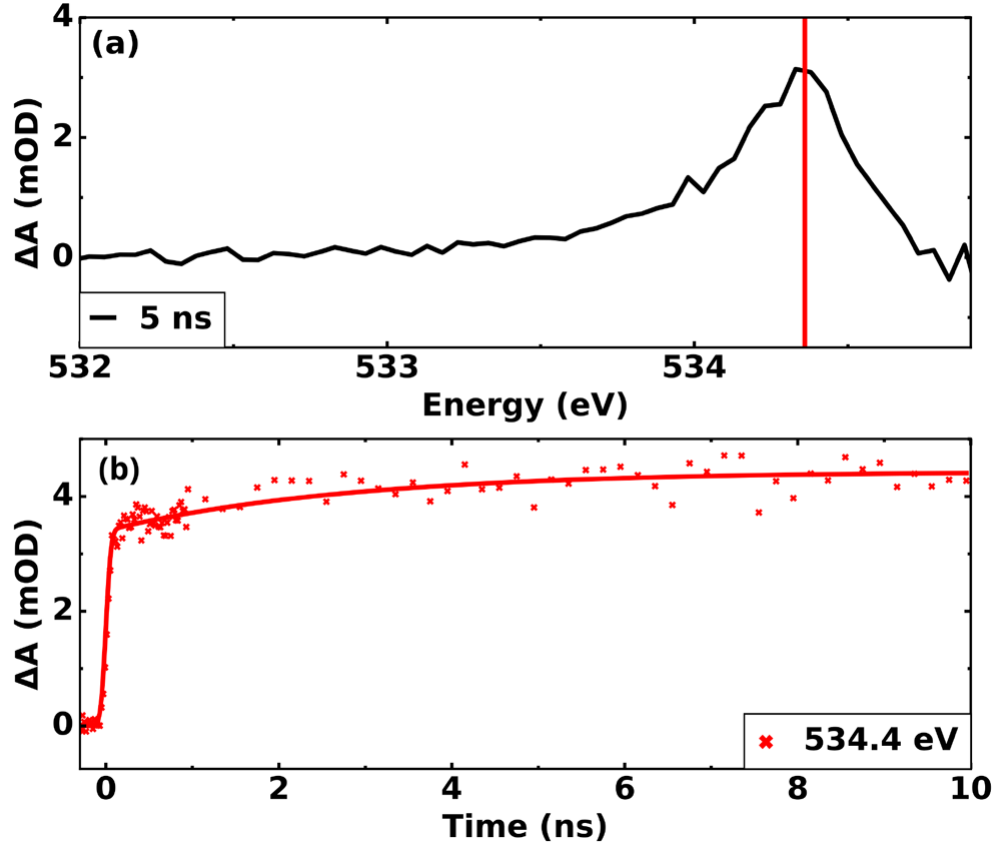
(a) Transient spectra of K_3_Co(CN)_6_ in water solution at the K-edge measured with a 5 ns time delay. (b) Delay trace measured at 534.4 eV. The increase in absorption below the water pre-edge, which grows with time, is a direct signature of the photoaquated species [Co(CN)_5_(H_2_O)]^2–^.

Looking at the transient data for the O K-edge shown in Figure 5a, the broad peak seen is attributed to the aquated photoproduct. Because the only O-bearing moiety in the product is the incoming water molecule, no more peaks were observed. Since the peak lies just before the pre-edge of water, we can assign it to the creation of the relatively stable aquated [Co(CN)_5_(OH_2_)]^2–^ as similarly reported by Vaz da Cruz et al.^13^ for [Fe(CN)_6_]^4–^.^13^ In Figure 5b the delay trace measured at 534.4 eV shows an analogous time-delay dependence as seen for the delay trace shown in Figure 4b measured at the 781.1 eV. Both signatures can be assigned to the aquated photoproduct, which confirms the formation of this species.

All of the pump–probe delay-dependent intensities (Figures 3b, 4b, and 5b) were fitted to a three-state kinetic model with two decay rates. The fit is represented by solid lines in Figures 3b, 4b, and 5b. The model yielded a time constant of 2.8 ns, to account for the triplet state intermediary, plus an exponential rise term, which yielded a time constant of 5.6 ns, to account for the stable and long-lived photoproduct. The instrument response function yielded 110.9 ps (fwhm) in a Gaussian profile fit. Details of the kinetic model and further parameters acquired by the global fit are presented in the Supporting Information.

The most recent studies on the [Fe(CN)_6_]^3–^^10,12,13^ point to a time scale of <20 ps for the formation of the aquated species. In a recent time-resolved XAS study at the metal L_3_-edge of the photoaquation of [Fe(CN)_6_]^4–^ reported by Vaz da Cruz et al.,^13^ it was noticed that the pre-edge region of the metal L-edge is sensitive to many of the possible intermediary states of the photoaquation reaction. At the temporal resolution of the order of ∼100 ps in the experiment, however, no indications of the triplet or any other intermediary species were found. This raises a question of why is the triplet state much shorter-lived in iron(II) than in cobalt(III), as well as why is the triplet state more reactive in iron than in cobalt. Given the detection of the ^3^T_1g_ state of [Co(CN)_6_]^3–^ as a transition state in the photoaqueous reaction, preliminary computations were performed in order to clarify these questions.

A look into how the states differ in each complex indicates how the state might also be responsible for the suppressed formation of aquated species seen in the cobalt(III) case. Rigid coordinate scans at the DFT level of theory are presented in Figure 6 for [Fe(CN)_6_]^4–^ and [Co(CN)_6_]^3–^ systems. It can be seen that when the triplet states of both metal complexes are compared, the cobalt(III) complex has a quasi-bound nature, while the iron(II) counterpart has a dissociative nature. This is in agreement with the measured lifetimes of these states. While the triplet state of iron dissociates on a time scale of a few picoseconds,^11^ it was shown by Conti et al.^19^ that the emission band attributed to the ^3^T_1g_ state of [Co(CN)_6_]^3–^ decays with a lifetime of 2.6 ns.^19^ In our measurements, a global fit of the delay traces yielded the decay time of 2.8 ns, which is in good agreement with Conti et al.^19^ and strengthens the hypothesis of an intermediary triplet state. Further computational details regarding the rigid coordinate scan can be found in the Supporting Information.

**Figure 6:**
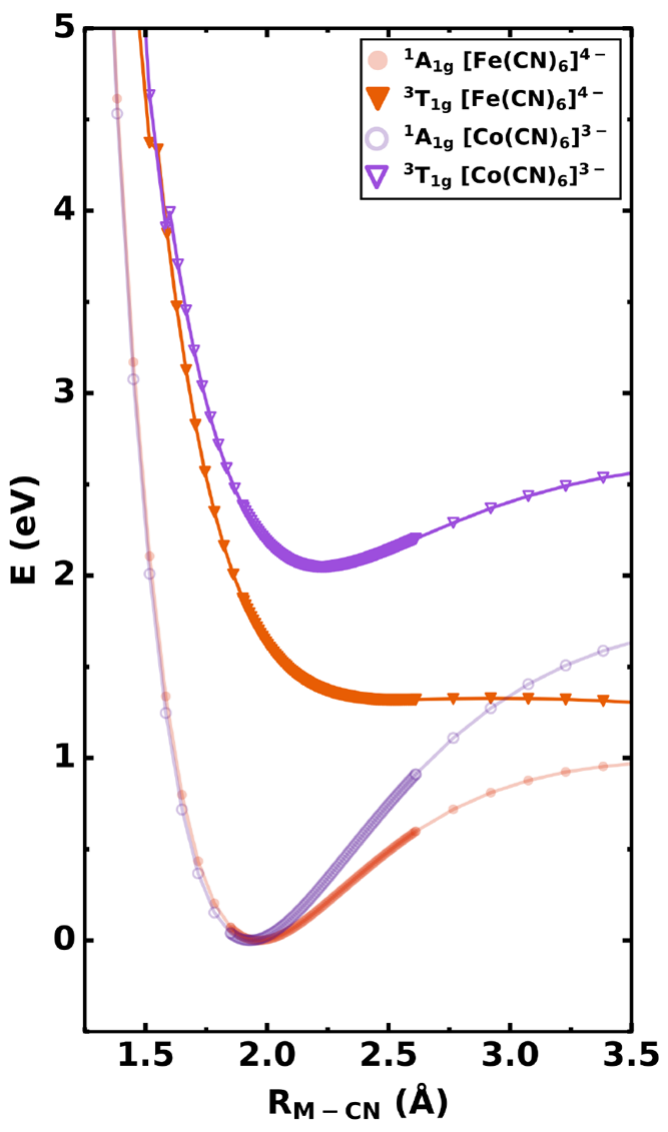
Potential energy curves computed for a rigid coordinate scan of the M-CN bond comparing the isoelectronic [Co(CN)_6_]^3–^ and [Fe(CN)_6_]^4–^ complexes. In both systems, ^1^A_1g_ is the ground state and ^3^T_1g_ is the lowest excited triplet. The computations were performed at the DFT level of theory employing the B3LYP^42^ functional and the def2-TZVP(-f) basis set.^32^ Solvation effects were considered with the conductor-like polarizable continuum model.^33^ Dispersion forces were taken into account via the D3^34^ correction using the Becke–Johnson damping model^35^ as implemented in the ORCA package.^31^

To explain the different nature of their first excited triplet states, we carried out a detailed investigation of the bonding channels in both complexes. A charge decomposition analysis^43,44^ (CDA) was performed, and it showed that, as the relative intensity of the satellite peak observed in each metal L_3_-edge indicates, the iron(II) hexacyanide presents higher admixture of ligand-centered π-acceptor orbitals when compared to the cobalt(III) counterpart, which in turn presents a higher degree of σ interaction with the ligands. Upon LF excitation, prior to intersystem crossing, an electron is removed from the metal centered t_2g_ set of orbitals and promoted to the unoccupied set of antibonding metal-centered e_*g*_ orbitals, thus effectively reducing the degree of back-bonding stabilization in the complex. In the iron(II) complex, the t_2g_ set of orbitals showed 10.5% π-acceptor character, in opposition to 2.9% in the cobalt(III) hexacyanide. This LF excitation, therefore, is expected to cause a stronger destabilization in [Fe(CN)_6_]^4–^ than in [Co(CN)_6_]^3–^. We believe this is a significant reason for why the first excited triplet state is dissociative in the iron(II) complex while it is weakly bound in the cobalt(III) counterpart. Further details of the CDA analysis can be found in the Supporting Information.

In this study, the photoaquation of [Co(CN)_6_]^3–^ was studied by time-resolved X-ray absorption spectroscopy at three complementary absorption edges, the Co L-, N K-, and O K-edges. The acquired data was interpreted in light of the mechanism proposed by Scandola^16^ and by means of time-dependent density functional theory calculations. Our transient X-ray absorption measurements show strong evidence of a long-lived triplet state intermediate via isolated spectral signatures related to the vacant t_2g_ orbital. Analysis of the temporal evolution of these signatures yielded a triplet state lifetime of 2.4 ns, in good agreement with reported data.^19^ The [Co(CN)_5_(OH_2_)]^2–^ photoaquated species was also unambiguously detected through distinct features associated arising from the Co–OH_2_ chemical bond, as well as a distinct temporal dependence differing from that of the triplet excited [Co(CN)_6_]^3–^.

From our results, a rather distinct picture arose for the photoaquation dynamics in cobalt(III) hexacyanide in comparison to the isoelectronic iron(II) hexacyanide.^13^ Namely, a much longer-lived triplet state is detected in [Co(CN)_6_]^3–^ with associated lower photoaquation yields for the cobalt-centered complex.^16^ We explain this discrepancy based on rigid coordinate scans of the metal–ligand bond calculated with density functional theory, which show that the excited triplet state in [Fe(CN)_6_]^4–^ is dissociative, whereas it is weakly bound for [Co(CN)_6_]^3–^. Conversely, photoaquation is suppressed due to the quasibound nature of the transient triplet state in the cobalt case. In the iron(II) complex, the intersystem crossing from ^1^T_1g_ to ^3^T_1g_ has a higher quantum efficiency, and the triplet intermediary state shows a complete dissociative character that contributes to the assured dissociation.^13^ The ^3^T_1g_ state of [Co(CN)_6_]^3–^, on the other hand, might favor relaxation to the ground state due to its less dissociative character than its Fe counterpart. From an analysis of the bonding-channels in both systems, we find indications that the dissociative character of this curve is inversely linked to the degree of back-bonding in the complex.

Lastly, we believe that future ultrafast transient X-ray measurements on these systems could help elucidate the details of the dissociative-bound character of the intermediate triplet state, as well as the branching ratio between photoaquation and possible vibrational relaxation to the minimum of a bound triplet state in [Co(CN)_6_]^3–^.

## Experimental Section

Experimental and computational details can be found in the Supporting Information.

## Supporting Information Available

Experimental procedures as well as computational methods and further information about the work (PDF)
Transparent Peer Review report available (PDF)
