# Supplementary material for: The Role of the Lowest Excited Triplet State in Defining the Rate of Photoaquation of Hexacyanometalates
Source: J Phys Chem Lett. 2024 Jan 2;15(1):241–7. doi: 10.1021/acs.jpclett.3c02775 (PMC10788954; doi:10.1021/acs.jpclett.3c02775)
Supplement: Supplementary file 1 — jz3c02775_si_001.pdf [file jz3c02775_si_001.pdf]

Supporting Information: The Role of the Lowest Excited Triplet State in Defining the Rate of Photoaquation of Hexacyanometalates

Eric J. Mascarenhas,[*,†] Mattis Fondell,[†] Robby Büchner,[†] Sebastian Eckert,[†] Vinicius Vaz da Cruz,[*,†] and Alexander Föhlisch[†,‡]

†*Institute Methods and Instrumentation for Synchrotron Radiation Research, Helmholtz-Zentrum Berlin für Materialien und Energie GmbH, 12489 Berlin, Germany*

‡*Institute of Physics and Astronomy, Universität Potsdam, 14476 Potsdam, Germany*

E-mail: eric.mascarenhas@helmholtz-berlin.de; vinicius.vaz_da_cruz@helmholtz-berlin.de

Experimental

$K_3Co(CN)_6$ was obtained from Santa Cruz Biotechnology and used without further purification. The salt was dissolved at a concentration of 200 mM in deionized water.

The experiments were carried out at the UE52-SGM[1] beamline at the synchrotron BESSY II. With the nmTransmission NEXAFS end station,[2] the liquid sample is transferred through two nozzles into the experimental vacuum chamber. When the two jets collide, a thin liquid sheet is created in which the thickness can be adjusted by changing the flow rate of the HPLC pump, or nozzle diameter. The static spectra in the region of the N K-edge and of the Co $L_{3,2}$-edge were acquired with 80 and 250 meV bandwidth of the incoming radiation, respectively, using a gallium arsenide photodiode, while the transient spectra were recorded with 55 and 175 meV bandwidth, respectively, with a silicon avalanche photodiode. The transient spectrum at the O K-edge was recorded with 90 meV bandwidth. The photodiode was equipped with a 200 nm Al filter in front of it. The photoexcitation was induced by the use of the 3rd harmonic (343 nm) of a fiber laser system with a fundamental wavelength of 1030 nm. The dynamics at the N K-edge were measured with this laser beam focused to a (60×80) μm^2 full width at half maximum (FWHM) spot with a pulse energy was 6.7 μJ, and fluence of 140 $mJ\cdot cm^{-2}$. For dynamic measurement at the Co $L_{3,2}$- as well as O K-edge, the laser spot size was (60×90) μm^2 FWHM, pulse energy was 13.9 μJ, and the laser fluence, 260 $mJ\cdot cm^{-2}$. More details of the experimental setup can be found in Fondell et al..[2] Supplemental measurements were made with the EDAX[3] endstation at the UE49-SGM[4] beamline at BESSY II.

Computation

All theoretical calculations were performed using the ORCA package.[5]

Structure Optimization

Structure optimization was performed at the density functional theory (DFT) level using the B3LYP[6] functional and the basis set def2-TVZP with the auxiliary basis set def2/J[7] in the RIJCOX approximation of ORCA[5] and with the D3[8] correction and the Becke-Johnson damping model for accounting for dispersion forces (D3BJ).[9] The Solvation effect was taken into account approximately by the conductor-like polarizable continuum model (CPCM)[10] for water. Optimized atomic positions in Angstroms used for computation of the x-ray absorption spectra are presented in tables 1-3.

Table 1: Atomic positions of $[Co(CN)_6]^{3-}$ in Angstroms optimized in DFT with B3LYP functional for computation of the N K-, and Co $L_{3,2}$-edges x-ray absorption computations.

Atom	x(Å)	y(Å)	z(Å)
C	-0.15179	-1.61784	1.95015
N	-1.01172	-2.39391	2.02118
Co	1.28281	-0.33782	1.81776
N	2.96577	-2.39359	0.24614
C	2.33378	-1.62024	0.83727
N	3.57733	1.71828	1.61434
C	2.71740	0.94220	1.68538
N	-0.40016	1.71796	3.38937
C	0.23183	0.94461	2.79825
N	0.10349	0.71174	-0.83544
C	0.54775	0.31831	0.16192
N	2.46213	-1.38739	4.47096
C	2.01786	-0.99395	3.47360

X-Ray Absorption Spectra

Spectral data were calculated at the time dependent density functional theory (TD-DFT)[11] level using the same functional and basis sets used for the optimization. All the spectra shown in the main paper were calculated with valence triple-zeta basis set, def2-TZVP(-f) with def2/J auxiliary basis set,[7] as implemented in ORCA, and the B3LYP functional.[6] Furthermore, to correct for dispersion error in the DFT level of theory, the D3BJ correc-

Table 2: Atomic positions of the $^3T_{1g}$ $[Co(CN)_6]^{3-}$ in Angstroms optimized in DFT with B3LYP functional for computation of the N K-, and Co-$L_{3,2}$-edges x-ray absorption computations.

Atom	x(Å)	y(Å)	z(Å)
C	-0.37239	-1.81086	1.97196
N	-1.22491	-2.59856	2.03522
Co	1.28285	-0.33778	1.81774
N	3.00282	-2.45010	0.20822
C	2.37307	-1.67516	0.79737
N	3.79054	1.92289	1.60060
C	2.93796	1.13524	1.66358
N	-0.43738	1.77440	3.42717
C	0.19248	0.99947	2.83811
N	0.10467	0.71256	-0.83905
C	0.54791	0.31970	0.15704
N	2.46105	-1.38818	4.47450
C	2.01779	-0.99528	3.47843

Table 3: Atomic positions of the photoaquated product $[Co(CN)_5(OH_2)]^{2-}$ in Angstroms optimized in DFT with B3LYP functional for computation of the N K-, and Co $L_{3,2}$-edges x-ray absorption computations.

Atom	x(Å)	y(Å)	z(Å)
Co	-0.31076	-0.07612	0.05369
C	-2.22792	-0.06805	-0.08620
C	1.60575	-0.06921	0.20009
C	-0.31041	-1.94030	0.02852
C	-0.45447	-0.10863	1.97165
C	-0.16724	-0.04988	-1.86462
O	-0.31715	1.98320	0.09885
H	-0.94474	2.32825	-0.55372
H	0.54817	2.32141	-0.17639
N	-0.30997	-3.09870	0.01064
N	-0.54154	-0.12335	3.12737
N	-0.08130	-0.01551	-3.02001
N	-3.38364	-0.04836	-0.17185
N	2.76123	-0.04224	0.28727

tion available in the computation package was used.[8,9] Due to the self-interaction error and core–hole relaxation effects,[12,13,13–15] the calculated spectra were shifted by 10.8 eV at the Co L_3 edge and 12.2 eV at the N K-edge to correspond to the observed experimental features.

The orbital excitation window was restricted to contain excitation from the 2p orbitals of Co for the Co $L_{3,2}$-edge and the 1s orbitals of the nitrogen atoms for the N K-edge spectrum. Spin-orbit coupling was included only in the computation shown with the steady state experimental spectra for completeness and was not considered in the time-dependent analysis in order to consistently compare closed-shell and open-shell species, since the TD-DFT computation for the later is not available in the ORCA package. Furthermore, The computed spectra were broadened with a Voigt profile with Lorentzian FWHM of 0.43 eV for the Co $L_{3,2}$-edge and 0.13 eV for the N K-edge to account for the core–hole lifetimes of each edge. The Gaussian part of the Voigt profile has a FWHM of 0.55 eV.

Rigid Coordinate scan

Data presented in Fig. 6 of the main text were calculated using the rigid coordinate scan capability of the ORCA package[5] in the DFT level of theory with def2-TZVP(-f) basis set, and def2/J auxiliary basis set[7] as well as D3BJ dispersion correction.[8,9] The same parameters were used for the results shown in Fig. 1. Rigid coordinate scan consist of a single point energy evaluation over a coordinate system. In our study, we evaluated the single point energy in function of the distance between the metal center and an axial CN^- ligand. Four functionals were studied: B3LYP,[6] BLYP,[16,17] BHandHLYP,[18] and CAM-B3LYP.[19] At the DFT level of theory, it is well known that the choice of functional has to be carrefully assessed in order to evaluate the physical liability of the conclusions drawn. Each of those functionals has its strengths and limitations,[20] but a clear qualitative trend was observed in the computations. The first type of functional to be developed were the ones based in the so called Local-Density Approximation (LDA).[21] As in this approximation the density is assumed to be the same everywhere, the description acquired with this type of functionals is

usually qualitative at the best. Fig. 1 shows for the computation using the BLYP functional, which is the simplest functional used in this study. For this computation, we see that the rigid scan for the two systems studied might behave very similarly. However, it is well known that LDA can be further improved by expanding the exchange-correlation functional in terms of the gradient of the density and some form of inhomogeneity is added to the problem. Furthermore, more specifically to the part of exchange, a component containing the computed Hartree-Fock (HF) exchange energy added along with the computed DFT one has shown to mitigate the errors seen in DFT computations. Functionals with some sort of HF component added to the exchange part are termed hybrid functionals.[21] In Fig. 1,

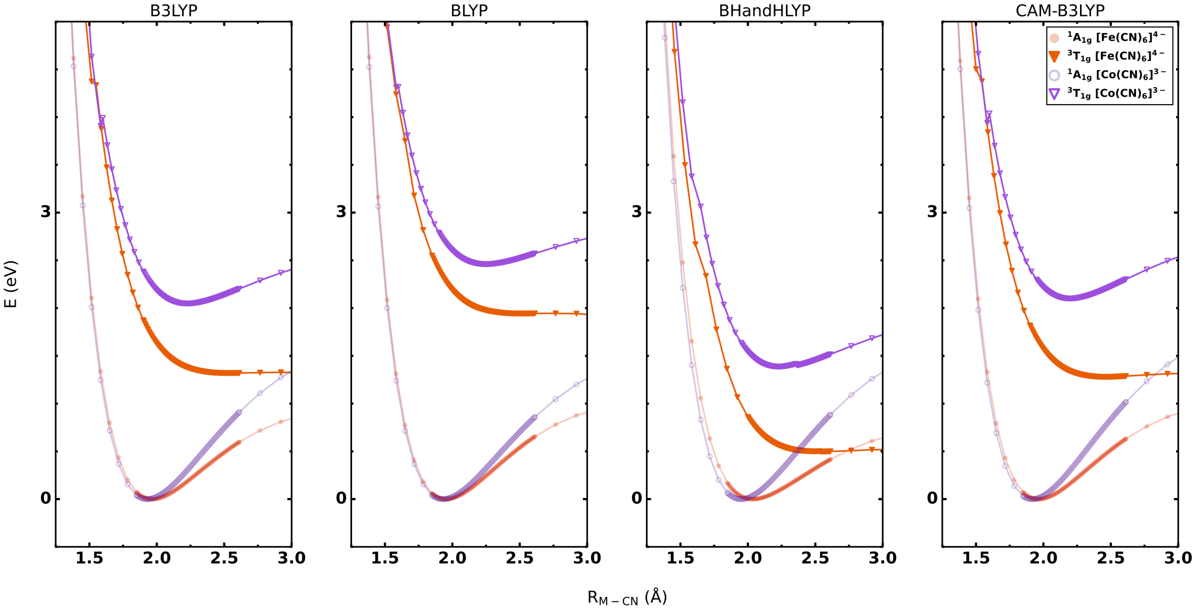

Figure 1: Rigid coordinate scan comparing $[Co(CN)_6]^{3-}$ and $[Fe(CN)_6]^{4-}$. In each of the computations the conductor-like polarizable continuum model[10] was used to account for the solvation environment and the D3[8] model with Becke-Johnson damping[9] was used for the correction of dispersion forces

the B3LYP based functionals contain 20% of HF exchange, while the BHandHLYP contains 50% of the same. In the CAM-B3LYP, this HF term is added with a soft function that distributes DFT and HF exchange energies accordingly to the spatial distance between two points r_{12} as described by Yanai et al.. In the systems studied here, the lowest excited

triplet showed to have a quasi bound nature in all the functionals when analysed along the M-L axis. The lowest triplet of the Fe complex showed a dissociative character for all the functionals studied. The results show a clear trend of distinction between the triplet state characteristic in both complexes.

Charge Decomposition Analysis

To shed light on the differences in bonding between $[Fe(CN)_6]^{4-}$ and $[Co(CN)_6]^{3-}$ we carried out a fragment charge decomposition analysis (CDA). In this way, we decompose the molecular orbitals of the complex in terms of those of the metal center and the cyanide ligands. Note that to minimize unphysical values, due to the Mulliken partition, we adopted the smaller def2-SV(P) basis set in this analysis. The complexes are isoelectronic with a d^6 configuration and belong to the O_h point group. The ligands have orbitals both in σ and π symmetry, which mix with the d orbitals of the metal ion, thereby we can, at least qualitatively, quantify the established bonding channels for this class of systems, namely σ-bonding, π-bonding and π^*-backbonding. The fragments chosen are composed by the $(CN)_6$ ligand system σ (donor), σ^* (acceptor), π (donor), and π^* (acceptor) orbitals, along with the free metal cation. The ligand orbitals with π symmetry mix with the t_{2g} set of metal d orbitals, whereas the ligand orbitals with σ symmetry mix with the metal e_g set of d orbitals. The results of the decomposition are shown in Fig. 2 along with an orbital diagram and plots of the orbitals analyzed.

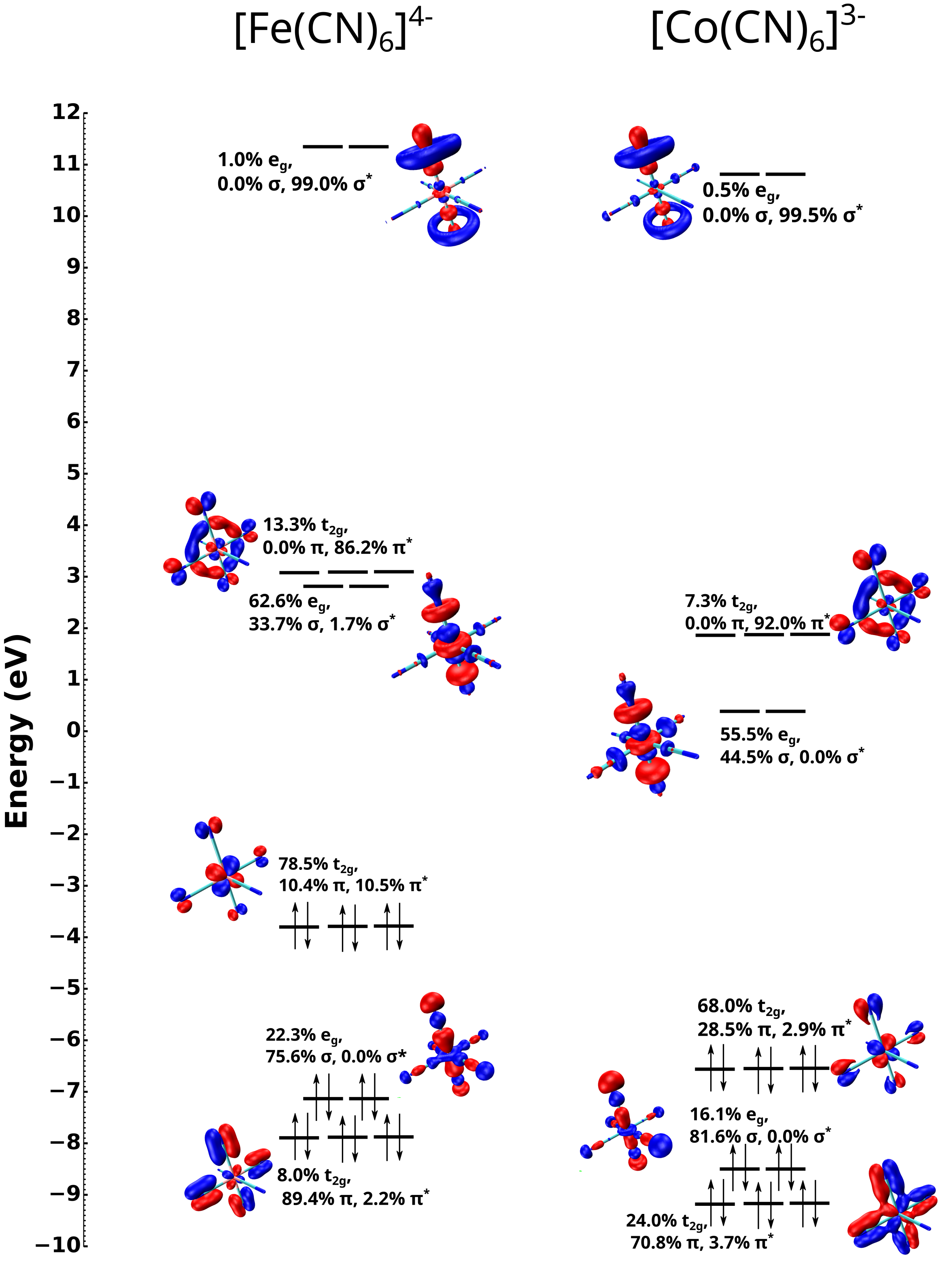

Figure 2: Kohn-Sham orbital diagram for $[Fe(CN)_6]^{4-}$ and $[Co(CN)_6]^{3-}$ showing the results of the fragment charge-decomposition analysis for selected molecular orbitals.

Details of the Kinetic Model and Fitting of Delay Traces

The time-resolved data was modelled by a three-states kinetic model consisting of the triplet state $^3T_{1g}$, the photoaquated species, here referred with index "aq", and the ground state $^1A_{1g}$.

$$\dot{\xi}_{^3T_{1g}}(t) = -\kappa_{^3T_{1g}}\xi_{^3T_{1g}},$$
$$\dot{\xi}_{aq}(t) = \kappa_{aq}\xi_{^3T_{1g}},$$
$$\dot{\xi}_{^1A_{1g}}(t) = (\kappa_{^3T_{1g}} - \kappa_{aq})\xi_{^3T_{1g}} \quad (1)$$

The system of equations is solved numerically with 10 ps time steps. The initial conditions for the populations of the triplet state and for the population of the aquated species was optimized and the following values were obtained: $\xi_{^3T_{1g}}(0) = 0.4$ and $\xi_{aq}(0) = 0.6$. The initial condition for population of the ground state was set to $\xi_{^1A_{1g}}(0) = -1$. The obtained time-dependent populations were convoluted with a Gaussian function with FWHM of 110.9 ps.

In total 8 delay dependent curves were experimentally acquired at the following photon energies: 399.5, 399.8, 777.6, 780.0, 781.1, 782.1, 784.6, and 534.4 eV.

Attempts to perform a global fit of all delay-traces were difficult, since there are several overlapping spectral signatures, arising from the three species. This led to the high correlation of many parameters, especially for the ground state bleaches in the Co L_3-edge and for the signals in the region of the N K-edge. Therefore, to obtain robust estimates of the lifetime constants we performed a reduced fit focusing on the isolated signal of the triplet state (777.6 eV), the isolated signal of the photo-aquated species (at the O K-edge) and the most intense ground-state bleach (782.1 eV). This procedure allowed us to extract the lifetime constants, and these values were fixed in the subsequent global fit which describes the full set of measured data. The lifetime of the triplet state and the rise time of the aquated species can be calculated as:

$$\tau_{^3T_{1g}} = \frac{1}{\kappa_{^3T_{1g}}},$$
$$\tau_{aq} = \frac{1}{\kappa_{aq}} \quad (2)$$

The values obtained were $\tau_{^3T_{1g}} = 2.8 \pm 0.2$ ns and $\tau_{aq} = 5.6 \pm 0.6$ ns. The values obtained for the amplitudes are summarized in Tab. 4. Unreported uncertainties could not be estimated due to the high correlation among the contributing amplitudes for the particular curve.

Table 4: Delay traces fit parameters. $A_{^3T_{1g}}$ stands for the triplet state amplitude, A_{aq} stands for the aquated species amplitude, and $A_{^1A_{1g}}$ stands for the ground state amplitude. Unreported uncertainties could not be estimated due to high correlation between the contributing components

Energy	$A_{^3T_{1g}}$	A_{aq}	$A_{^1A_{1g}}$
399.5	1.00E-01	9.78E-02	9.74E-02
399.8	1.17E-02	1.33E-02	1.34E-02
776.6	1.63E-03±6.0E-05		
780.0	2.47E-03±9.0E-05	4.20E-04±3.0E-05	
781.1	2.16E-03	2.09E-03	1.34E-03
782.1	2.60E-06	8.01E-04	2.13E-03
784.6	9.82E-04	1.62E-03	2.43E-03
534.4		5.52E-03±2.0E-05	

Close-up at the N K-edge transient spectrum

Fig. 3a of the main text presents the transient spectra of $[Co(CN)_6]^{3-}$ after photoexcitation by a 343 nm laser. The pre-edge region that spans from 395.0 eV to 398.5 eV presents small oscillations. In Fig 3c we show the computed spectra of the $[Co(CN)_6]^{3-}$ excited to its lowest excited triplet state with label $^3T_{1g}$, and also of the photoproduct, $[Co(CN)_5(OH_2)]^{2-}$. Plotting the experimental transient spectrum along with the theoretical x-ray absorption computation, shows that the oscilations seen in the pre-edge region might be features pointing to the species involved in the photoaquation. We show this plot with a zoom in the pre-edge

region in Fig. 3d, binning the experimental data for better clarity. The version without binning is presented in Fig. 3.

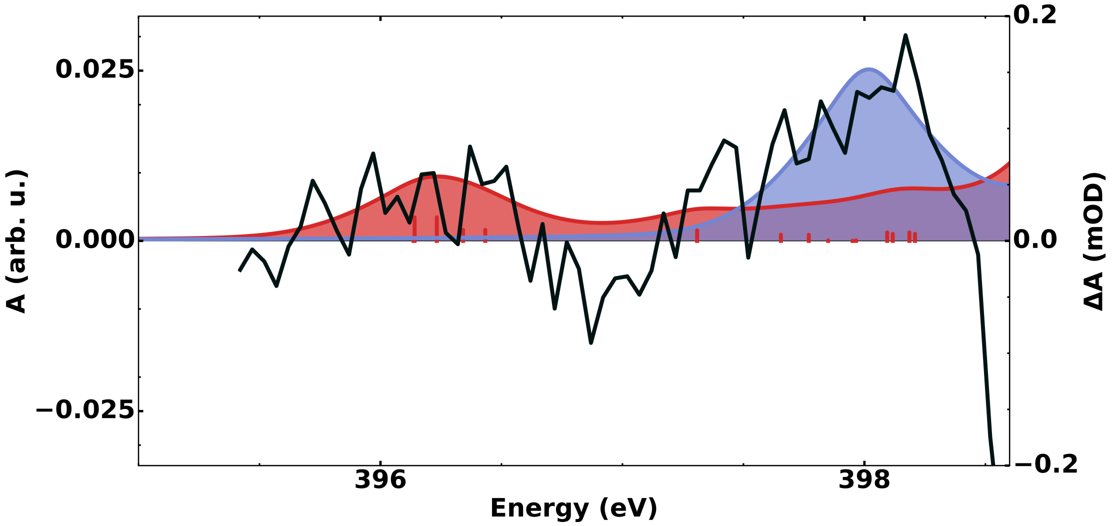

Figure 3: Data presented in the Fig. 3d of the main text without binning factor

Effect of the pump laser on the solvent (water)

The spectrum shown in Fig. 5a of the main text is presented in 4 along with the x-ray absorption spectrum of pure water. It is possible to see that the transient peak lies ≈ 0.6 eV below the pre-edge of water. The pre-edge of water is atributed to electronic transition from the 1s orbital of oxygen to the 4_{a1} molecular orbital of water.[22] Previous studies of photoaquation have seen that this peak shifts upon coordination with water,[23] what we indeed observe in 4. To rule out any effect of warming in the solvent, a transient measurement was performed in the pure solvent and no feature was found. The transient measurement of the pure solvent under laser is also shown in 4.

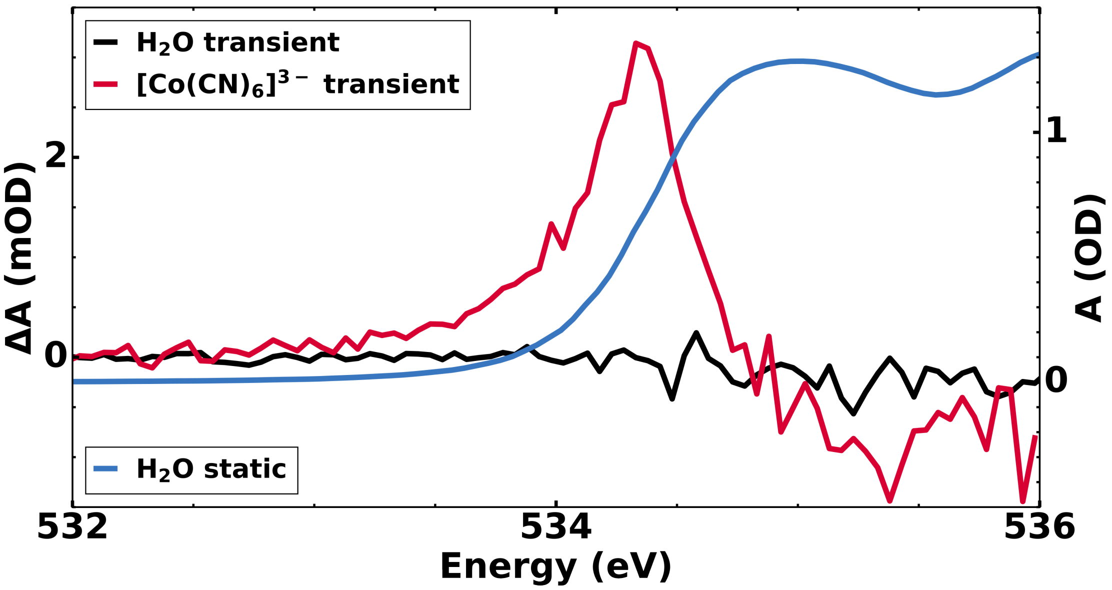

Figure 4: Comparison showing our transient after pumping, pure water after pumping, and the static spectrum of water showing the pre-peak feature of water for reference. The pump pulse has 343 nm wavelength

References

(1) Miedema, P. S.; Quevedo, W.; Fondell, M. The Variable Polarization Undulator Beamline UE52 SGM at BESSY II. *Journal of large-scale research facilities JLSRF* **2016**, *2*, 70.

(2) Fondell, M.; Eckert, S.; Jay, R. M.; Weniger, C.; Quevedo, W.; Niskanen, J.; Kennedy, B.; Sorgenfrei, F.; Schick, D.; Giangrisostomi, E. et al. Time-Resolved Soft X-ray Absorption Spectroscopy in Transmission Mode on Liquids at MHz Repetition Rates. *Structural Dynamics* **2017**, *4*, 054902.

(3) Kunnus, K.; Rajkovic, I.; Schreck, S.; Quevedo, W.; Eckert, S.; Beye, M.; Suljoti, E.; Weniger, C.; Kalus, C.; Grübel, S. et al. A Setup for Resonant Inelastic Soft X-Ray Scattering on Liquids at Free Electron Laser Light Sources. *Review of Scientific Instruments* **2012**, *83*, 123109.

(4) Pietzsch, A.; Eisebitt, S. The UE49 SGM RICXS Beamline at BESSY II. *Journal of large-scale research facilities JLSRF* **2016**, *2*, 54.

(5) Neese, F. Software Update: The ORCA Program System—Version 5.0. *WIREs Computational Molecular Science* **2022**, *12*, e1606.

(6) Stephens, P. J.; Devlin, F. J.; Chabalowski, C. F.; Frisch, M. J. Ab Initio Calculation of Vibrational Absorption and Circular Dichroism Spectra Using Density Functional Force Fields. *J. Phys. Chem.* **1994**, *98*, 11623–11627.

(7) Weigend, F.; Ahlrichs, R. Balanced Basis Sets of Split Valence, Triple Zeta Valence and Quadruple Zeta Valence Quality for H to Rn: Design and Assessment of Accuracy. *Physical Chemistry Chemical Physics* **2005**, *7*, 3297.

(8) Grimme, S.; Antony, J.; Ehrlich, S.; Krieg, H. A Consistent and Accurate Ab Ini-

tio Parametrization of Density Functional Dispersion Correction (DFT-D) for the 94 Elements H-Pu. *The Journal of Chemical Physics* **2010**, *132*, 154104.

(9) Grimme, S.; Ehrlich, S.; Goerigk, L. Effect of the Damping Function in Dispersion Corrected Density Functional Theory. *Journal of Computational Chemistry* **2011**, *32*, 1456–1465.

(10) Barone, V.; Cossi, M. Quantum Calculation of Molecular Energies and Energy Gradients in Solution by a Conductor Solvent Model. *The Journal of Physical Chemistry A* **1998**, *102*, 1995–2001.

(11) Runge, E.; Gross, E. K. U. Density-Functional Theory for Time-Dependent Systems. *Physical Review Letters* **1984**, *52*, 997–1000.

(12) DeBeer George, S.; Petrenko, T.; Neese, F. Prediction of Iron K-Edge Absorption Spectra Using Time-Dependent Density Functional Theory. *The Journal of Physical Chemistry A* **2008**, *112*, 12936–12943.

(13) Tu, G.; Carravetta, V.; Vahtras, O.; Ågren, H. Core Ionization Potentials from Self-Interaction Corrected Kohn-Sham Orbital Energies. *The Journal of Chemical Physics* **2007**, *127*, 174110.

(14) Besley, N. A.; Gilbert, A. T. B.; Gill, P. M. W. Self-Consistent-Field Calculations of Core Excited States. *The Journal of Chemical Physics* **2009**, *130*, 124308.

(15) Hait, D.; Head-Gordon, M. Highly Accurate Prediction of Core Spectra of Molecules at Density Functional Theory Cost: Attaining Sub-electronvolt Error from a Restricted Open-Shell Kohn–Sham Approach. *The Journal of Physical Chemistry Letters* **2020**, *11*, 775–786.

(16) Becke, A. D. Density-Functional Exchange-Energy Approximation with Correct Asymptotic Behavior. *Phys. Rev. A* **1988**, *38*, 3098–3100.

(17) Lee, C.; Yang, W.; Parr, R. G. Development of the Colle-Salvetti Correlation-Energy Formula into a Functional of the Electron Density. *Phys. Rev. B* **1988**, *37*, 785–789.

(18) Becke, A. D. A New Mixing of Hartree–Fock and Local Density-functional Theories. *J. Chem. Phys.* **1993**, *98*, 1372–1377.

(19) Yanai, T.; Tew, D. P.; Handy, N. C. A New Hybrid Exchange–Correlation Functional Using the Coulomb-attenuating Method (CAM-B3LYP). *Chemical Physics Letters* **2004**, *393*, 51–57.

(20) Burke, K.; Wagner, L. O. DFT in a Nutshell. *International Journal of Quantum Chemistry* **2013**, *113*, 96–101.

(21) Koch, W.; Holthausen, M. *A Chemist's Guide to Density Functional Theory*; John Wiley & Sons, Ltd, 2001; Chapter 6, pp 65–91.

(22) Niskanen, J.; Fondell, M.; Sahle, C. J.; Eckert, S.; Jay, R. M.; Gilmore, K.; Pietzsch, A.; Dantz, M.; Lu, X.; McNally, D. E. et al. Compatibility of Quantitative X-ray Spectroscopy with Continuous Distribution Models of Water at Ambient Conditions. *Proceedings of the National Academy of Sciences* **2019**, *116*, 4058–4063.

(23) Vaz da Cruz, V.; Mascarenhas, E.; Büchner, R.; M. Jay, R.; Fondell, M.; Eckert, S.; Föhlisch, A. Metal–Water Covalency in the Photo-Aquated Ferrocyanide Complex as Seen by Multi-Edge Picosecond X-ray Absorption. *Physical Chemistry Chemical Physics* **2022**, *24*, 27819–27826.
