# Supplementary material for: The Role of the Lowest Excited Triplet State in Defining the Rate of Photoaquation of Hexacyanometalates
Source: J Phys Chem Lett. 2024 Jan 2;15(1):241–7. doi: 10.1021/acs.jpclett.3c02775 (PMC10788954; doi:10.1021/acs.jpclett.3c02775)
Supplement: Supplementary file 2 — jz3c02775_si_002.pdf [file jz3c02775_si_002.pdf]

jz-2023-027753.R1

Name: Peer Review Information for "The Role of the Lowest Excited Triplet State in Defining the Rate of Photoaquation of Hexacyanometalates"

First Round of Reviewer Comments

Reviewer: 1

Comments to the Author

This article presents a nicely carried out steady-state and time-resolved X-ray absorption study of Co(CN)6. The quality of the data is remarkable and their interpretation is supported by TDDFT calculations. The authors have put forward convincing arguments. I recommend publication as is.

Reviewer: 2

Comments to the Author

This manuscript describes time-resolved X-ray absorption measurements at the Co(III) ligand K and metal L-edges of the complex [Co(CN)6]3- after photoexcitation of a ligand-field transition. A lot of the results are compared to calculations, but also to previous work by the same group on [Fe(CN)6]2-. The paper is quite hard to follow, but the main conclusion seems to be that the quantum yield of forming the photo-product replacing on CN- ligand with a water molecule, which it is claimed to proceed via a triplet state in both cases, is lower for [Co(CN)6]3- than for [Fe(CN)6]2- because of a minimum in the triplet state potential energy surface (PES) of [Co(CN)6]3- but a repulsive PES for [Fe(CN)6]2-.

The experimental results are presented in a quite obscure manner with areas under peaks coloured in, making it quite hard to separate the calculated and experimental spectra (mainly in Fig. 2). And sometimes they are smoothed, without giving any more details. Nevertheless, there are some serious experimental questions that needs to be answered if this work should be published. As far as I can see in the SI, there were no power dependence of the pump laser carried out. A value of 331mJ/cm2 is very high and can lead to thermal and/or non-linear effects. Since a plot of signal vs laser power has not been presented, I find it hard to gauge the reliability of the data. Furthermore, the concentrations were also quite high, and I would also like to see a dependence on the measured extinction coefficient vs concentration so check that there is no aggregation etc.

The motivation for the study should be made clearer. In the introduction, there is a lot of mentioning of earth-abundant photocatalysis etc, but why is the story about the triplet state in [Co(CN6)]2- actually relevant for the readers of J Chem Phys Lett? Seems like most of the recent literature cited in the introduction about this specific problem is from the 1960s. Is it the time-resolved XAS method that is adding to this problem? Or is it the finding of the minimum of the triplet state PES? It seems like it's a well-studied problem and the present paper does not really add anything (if my understanding is correct, it might add some contradicting conclusions regarding the involvement of the triplet state, which is interesting).

There are a few other issues I find confusing with the paper. If the triplet lifetime is an intermediate state in the photodissociation process, then why is there no growth of the photoproduct? A rise time of 54 ns is mentioned to be associated with the signal of the photoproduct (781.1 eV), but that is not seen in fig 4 b where the photoproduct signal is formed immediately. For the sake of the argument, if the triplet state is the doorway state for the photoproduct, then why are photoproducts forming with a rise time of 54 ns, when the measured lifetime of the triplet state (in this study) is only 2.4 ns? This is very confusing and does not seem right. To me, this implies that the photoproduct is formed more or less immediately and the triplet state is not involved at all in the process, in contrast to what is claimed in the paper. I wonder if the product peak (781.1eV in the L-edge but also in the O spectrum in fig 5) is just a result of solvent heating due to the high laser power? The O spectrum is also slightly strange as it must be hard to measure the photoproduct if it contains the same molecule as the solvent. Again, perhaps solvent heating? Fig 4 b needs to be replotted. It's very hard to see the data, plus an expanded version showing the 54 ns growth is needed to convince the reader about the growth.

A previous study on the triplet state lifetime is cited (ref 28) and they say it's also in the ns range. Please comment on the values you obtained and that previously measured (the reader was left quite confused by this one short sentence (page 14). Same comment for the sentence about the static spectra reported by Lalithambika et al. Are they similar to yours?

Are the calculated spectra shifted to match the experimental data? I guess this is OK since DFT might produce a constant mismatch that can be compensated. However, given that the static L-edge spectra Fig 2b do not agree to within several eV, why should we trust the calculated spectra of the triplet state and photoproducts to the level that they have been used to assigned the transient spectra in Fig 4c? The whole conclusion of the paper is based on this assignment and I find it quite hard to be convinced. Likewise, In Fig 4a and c, why are the calculated peaks from 786-788 eV not seen in the transient spectra? You rely heavily on assigning the peaks in the 776-780 eV region, but completely ignore the missing peaks in the 786-788 eV range.

What is the experimental evidence that "photoaquation is suppressed due to the quasibound nature of the transient triplet state"?

From the calculations in Fig 6, why is there a minimum in the Co(CN)6 triplet state PES but not in the Fe(CN)6 PES? Is there a way to rationalise this? I can see that there is no way for the triplet state in Co(CN)6 to decay but what about other reaction coordinates? Could there be conical intersections to other PES?

When you say a "smoothed version of the data…" please clarify what smoothing process has been applied and show what the unsmoothed data look like in the SI.

Overall, it might be that a rewritten version of the manuscript will make the arguments more clear, as I found the level of English to make it quite hard to follow (I will not comment specifically on the grammar, structure of paragraphs etc, but this should be considered much more carefully).

In the introduction, specify which complex has been studied, don't just say Fe(II) has been studied (I guess you're referring to Fe(bpy)3 etc).

I think it should be called "the triplet state", not just "the triplet". This is should also be implemented in the title of the paper.

Introduction: replace "spatial" with geometrical?

Co and Cobalt was used interchangeably in the manuscript. Please be consistent.

Author's Response to Peer Review Comments:

Reviewer(s)' Comments to Author:

Reviewer: 1

Recommendation: This paper represents a significant new contribution and should be published as is.

Comments:

This article presents a nicely carried out steady-state and time-resolved X-ray absorption study of Co(CN)6. The quality of the data is remarkable and their interpretation is supported by TDDFT calculations. The authors have put forward convincing arguments. I recommend publication as is.

We are very grateful to the reviewer for such an overwhelmingly positive evaluation of our work.

Reviewer: 2

Recommendation: This paper may be publishable, but major revision is needed; I would like to be invited to review any future revision.

Comments:

This manuscript describes time-resolved X-ray absorption measurements at the Co(III) ligand K and metal L-edges of the complex [Co(CN)6]3- after photoexcitation of a ligand-field transition. A lot of the results are compared to calculations, but also to previous work by the same group on [Fe(CN)6]2-. The paper is quite hard to follow, but the main conclusion seems to be that the quantum yield of forming the photo-product replacing on CN- ligand with a water molecule, which it is claimed to proceed via a triplet state in both cases, is lower for [Co(CN)6]3- than for [Fe(CN)6]2- because of a minimum in the triplet state potential energy surface (PES) of [Co(CN)6]3- but a repulsive PES for [Fe(CN)6]2-.

1) **The experimental results are presented in a quite obscure manner with areas under peaks coloured in, making it quite hard to separate the calculated and experimental spectra (mainly in Fig. 2). And sometimes they are smoothed, without giving any more details. Nevertheless, there are some serious experimental questions that needs to be answered if this work should be published. As far as I can see in the SI, there were no power dependence of the pump laser carried out. A value of 331mJ/cm2 is very high and can lead to thermal and/or non-linear effects. Since a plot of signal vs laser power has not been presented, I find it hard to gauge the reliability of the data. Furthermore, the concentrations were also quite high, and I would also like to see a dependence on the measured extinction coefficient vs concentration so check that there is no aggregation etc.**

We thank the referee for their comments. Regarding the presentation of the steady state data, we assessed your suggestion and reworked Fig. 2 without coloring the area under the curve. This way, we believe the computational results are better distinguished from the experimental. Furthermore, the only curve we smoothed was the one labeled as Fig. 3d. This square in the plot is a zoom of an almost flat region of Fig. 3a where the data does not seem of interest at first glance being plotted along with the strong main signal around 400 eV. Nevertheless, the smoothed data is presented along with the original measurement. We believe the features highlighted are better distinguished when we bin the data by a factor of 3. As the features are not the main hint on the dynamics, but corroborate to our model, we found more clarifying to present the smoothed curve in a zoomed in version as a highlight of the figure. We understand, however, the referee's confusion and we have made available the plot without binning in the supporting information.

Regarding the laser fluence, we recalculated the values and found that the presented fluence for the experiment at the Co L- and O K-edge, although of the same order, was incorrect. The values were recalculated and in both experiments the pulse energy of our 208 kHz laser system was in the order of ~ 10 µJ which is in agreement with former pump-probe experiments in systems similar to ours.

As for the concentration, the $K_3[Co(CN)_6]$ is a highly soluble salt with a maximum solubility in water on the order of 1 M. Previous experiments on similar systems have relied on a concentration ranging from 100 to 300 mM as in Huse *et al* J. Phys. Chem. Lett. (2011) 2, 8, 880-884, Jay *et al* J. Phys. Chem. Lett. (2018) 9, 12, 3538–3543, Vaz da Cruz *et al* Phys. Chem. Chem. Phys. (2022) 24 (45), 27819-27826, and Reinhard *et al* Nat. Commun. (2023) 14,2443. We should also point out that due to the typical metal $L_{3,2}$-edge cross section, and the small cross-section of photoexcitation in a forbidden d-d transition, we have seen that a typical ideal concentration for this type of experiment lies in the aforementioned range. In summary, the value used for concentration was chosen to match former pump-probe experiments in similar hexacyanometalate systems. It is also important to reiterate that for experiments in large scale facilities, like synchrotron light sources, it is not always possible to perform systematic physical chemical characterizations as the one suggested due to the limited beamtime granted by the facilities.

2) **The motivation for the study should be made clearer. In the introduction, there is a lot of mentioning of earth-abundant photocatalysis etc, but why is the story about the triplet state in [Co(CN6)]2- actually relevant for the readers of J Chem Phys Lett? Seems like most of the recent literature cited in the introduction about this specific problem is from the 1960s. Is it the time-resolved XAS method that is adding to this problem? Or is it the finding of the minimum of the triplet state PES? It seems like it's a well-studied problem and the present paper does not really add anything (if my understanding is correct, it might add some contradicting conclusions regarding the involvement of the triplet state, which is interesting).**

We have attempted to make the motivation of our study clearer and have rewritten our introductory paragraphs. We believe the reported data is of high interest to the readership of J. Phys. Chem. Lett. as it reports on an important light-driven physico-chemical transformation on a topical class of transition metal systems highly debated in the community and readership of J. Phys. Chem. Lett. Moreover, we cannot agree with the reviewer that the photo-aquation of $[Co(CN)_6]^{3-}$ is a well studied problem. Perhaps the reviewer has the pair of Iron-cyanide complexes in mind, which have been extensively subjected to modern time-resolved spectroscopy. The case of $[Co(CN)_6]^{3-}$ has in fact remained underexplored and is for the first time addressed in this article with state-of-the-art spectroscopic techniques. We are sorry to read that the reviewer holds the opinion that our study does not add anything to the question at hand, however we must politely disagree with such an evaluation. Although it is true that the existence of the triplet state was established in the 70s several details remained uncovered, not to mention the disconcerting question on the lower photo-aquation yield of $[Co(CN)_6]^{3-}$ when compared with its Iron counterpart. Our article presents very high-quality transient X-ray spectra measurements that reveal the existence of the triplet state with high sensitivity to the metal density of states with clear t_{2g}-hole signatures. These are supplemented by N K-edge measurements focused on the cyanide ligands as well as O K-edge measurements which are mostly sensitive to the aquated photo-product. These measurements are combined with density functional theory calculations to provide an explanation to the reduced quantum-yield of photoaquation in $[Co(CN)_6]^{3-}$ when compared with $[Fe(CN)_6]^{4-}$. We are thankful to the reviewer for requesting clarifications on this point, as in the revised manuscript we have

further analyzed our results and calculations to give a deeper insight into the origin of this fundamental problem.

3) **There are a few other issues I find confusing with the paper. If the triplet lifetime is an intermediate state in the photodissociation process, then why is there no growth of the photoproduct? A rise time of 54 ns is mentioned to be associated with the signal of the photoproduct (781.1 eV), but that is not seen in fig 4 b where the photoproduct signal is formed immediately. For the sake of the argument, if the triplet state is the doorway state for the photoproduct, then why are photoproducts forming with a rise time of 54 ns, when the measured lifetime of the triplet state (in this study) is only 2.4 ns? This is very confusing and does not seem right. To me, this implies that the photoproduct is formed more or less immediately and the triplet state is not involved at all in the process, in contrast to what is claimed in the paper. I wonder if the product peak (781.1eV in the L-edge but also in the O spectrum in fig 5) is just a result of solvent heating due to the high laser power? The O spectrum is also slightly strange as it must be hard to measure the photoproduct if it contains the same molecule as the solvent. Again, perhaps solvent heating? Fig 4 b needs to be replotted. It's very hard to see the data, plus an expanded version showing the 54 ns growth is needed to convince the reader about the growth.**

The delay trace curve shown in Fig. 4b (blue, 781.1 eV) was attributed mainly to photoproduct formation. Our computation has shown that this region most likely also overlaps with the tails of one of the signals that identifies the first triplet excited state. For this reason, the maxima seen at the 781.1 eV delay trace in Fig. 4b can not directly be attributed to the formation of only photoproducts, but also to the formation of the intermediary state. At longer delays, however, the signals attributed to the excited species decreases and the signal attributed to be the signature of the photoproduct remains constant. The same is not observed in the delay trace presented in Fig. 5b, which shows that the signal reaches its maximum over tens of nanoseconds. Furthermore, it is expected that the substitution of one CN^- molecule by one H_2O would yield one single peak in the transient spectrum, which we indeed observe. This corroborates to the formation of a short-lived excited species, and a long-lived product stable over at least the nanoseconds timescale.

We understand that a laser system could cause heating in the solvent and that might appear as a transient signal. Thinking of that, we excluded side effects of the laser system in the solvent by repeating the experiment in pure water. We added this analysis at the O K-edge in the supporting information for better clarification. There, we can see that the signal observed corresponds to a red shifted pre-peak of water, as expected for a H_2O molecule acting as ligand. Furthermore, the transient measurements have found no signal in the O K-edge of the pure solvent after exposure to the laser with the same parameters used in the analysis of the sample.

4) **A previous study on the triplet state lifetime is cited (ref 28) and they say it's also in the ns range. Please comment on the values you obtained and that previously measured (the reader was left quite confused by this one short sentence (page 14).**

Same comment for the sentence about the static spectra reported by Lalithambika et al. Are they similar to yours?

The study cited (ref. 28) is the work by Conti et al. in which the authors measure the lifetime of the triplet state by tracking over time the amplitude of the emission band attributed to the decay of this state. Conti investigates the stability of this state in various mixtures of polyalcohols and water. For pure water in the $[Co(CN)_6]^{3-}$ system, the author finds a value of 2.6 ns which lies in close agreement to the lifetime measured in our experiment. For the transition $^3T_{1g} – ^1A_{1g}$, it is expected that multiple deactivation channels exist, since the transition is forbidden by spin. From former electronic structure studies on d^6 hexacyanometalates, we know the possible states involved, namely the slightly higher lying $^3T_{2g}$, $^1T_{1g}$ or $^1T_{2g}$, and we can infer that the main contribution to the lifetime measured is derived from the lowest excited triplet state.

5) **Are the calculated spectra shifted to match the experimental data? I guess this is OK since DFT might produce a constant mismatch that can be compensated.**

Yes, the calculated spectra are shifted by 10.8 eV for the Co L_3-edge and 12.2 eV for the N K-edge ,respectively, to match the experiment as it's a known deficiency in TD-DFT calculations of X-ray spectra.

6) **However, given that the static L-edge spectra Fig 2b do not agree to within several eV, why should we trust the calculated spectra of the triplet state and photoproducts to the level that they have been used to assigned the transient spectra in Fig 4c? The whole conclusion of the paper is based on this assignment and I find it quite hard to be convinced. Likewise, In Fig 4a and c, why are the calculated peaks from 786-788 eV not seen in the transient spectra? You rely heavily on assigning the peaks in the 776-780 eV region, but completely ignore the missing peaks in the 786-788 eV range.**

The reviewer brings up an important aspect. To answer their question, it is necessary to bear in mind the interpretation of the L-edge absorption spectra of metal hexacyanides. Namely, the main peak is related to metal-centered unoccupied e_g orbitals and the second peak, as discussed in the seminal work of Hocking et al J.A.C.S. (2006), 128, 10442–10451, is related to π*-backbonding, which are mostly ligand-centered unoccupied states with admixture of metal t_{2g} orbitals. We emphasize that our calculations reproduce both of these features. However, as the reviewer correctly points out, the calculated energy position of the satellite peak is not an exact match to the experiment. The energy position of the satellite peak in the spectrum of cyanides is known to be difficult to calculate even for higher level theories (see the work of Kunnus *et al.* J. Phys. Chem. B (2016), 120, 29, 7182–7194 and Engel *et al.* J. Phys. Chem. B (2014), 118, 6, 1555–1563 where RASPT2 calculations are employed). The reason for this is mainly the cyanide-π*-metal-t_{2g} charge-transfer character, additionally this feature is affected by configuration interaction with the main e_g peak of the spectrum.

Notwithstanding, the qualitative spectral shape is very well reproduced and very good accuracy is obtained for the features around 786-788 eV, which are the main objects of our study, as these features

are the most sensitive to the d-orbital occupation as well as the ligand-substitution photo-reaction. The region of the transient spectrum around the 786-788 eV energy range, mentioned by the reviewer, is very made up of several overlapping transitions and is mostly dominated by a ground-state bleach, making it more ambiguous and less useful for spectroscopic fingerprinting, as opposed to the region around the main e_g peak in the 786-788 eV range.

Since this is an important and non-trivial aspect, we have included the discussion above in the revised version of the manuscript.

7) **What is the experimental evidence that "photoaquation is suppressed due to the quasibound nature of the transient triplet state"?**

The quantum yield for the photoaquation of $[Fe(CN)_6]^{4-}$ was reported to vary with wavelength and pH reaching a maximum of 0.89 as reported by Shirom and Stein in The Journal of Chemical Physics, 55, 3372-3378 (ref. 15). The quantum yield for photoaquation of $[Co(CN)_6]^{3-}$, on the other hand, is pH and wavelength independent with a value of 0.31 as reported by Porter in the Journal of the American Chemical Society, 91, 3980-3982 (ref. 26).

As opposed to our previous investigation of the photo-aquation in $[Fe(CN)_6]^{4-}$ (P.C.C.P 2022, **24**, 27819-27826), we found evidence for a long-lived triplet state intermediary, this fact alone implies a distinct dynamics of the triplet excited state in $[Co(CN)_6]^{3-}$, in line with the lower quantum-yields mentioned above. In order to give insight into the origin of such discrepancy, we carried out potential energy calculations along the Metal-CN bond stretching coordinate which indicate that the first excited triplet state has a stronger dissociative character in the iron centered hexacyanide than in the cobalt centered counterpart. Based on the combination of experimental and computational evidence, we propose the difference in the potential energy curve profile of the triplet excited state to be the leading factor for the suppressed photoaquation reaction in the cobalt hexacyanide complex.

8) **From the calculations in Fig 6, why is there a minimum in the Co(CN)6 triplet state PES but not in the Fe(CN)6 PES? Is there a way to rationalise this? I can see that there is no way for the triplet state in Co(CN)6 to decay but what about other reaction coordinates? Could there be conical intersections to other PES?**

The stability in both complexes can be attributed to a synergy between direct and back bonding. While the ligands donate electronic density through σ and π bonding channels, the back-bonding is almost entirely governed by π bonding channels. In this regard, The decomposition shows for the $[Fe(CN)_6]^{-4}$ bound levels a higher admixture of π acceptor orbitals. On the other hand, the bonding states of the cobalt complex have shown to present higher contributions from sigma bonding channels with overall higher electron delocalization. The removal of one electron from the HOMO t_{2g} set of the metal centered orbitals and addition to the σ e_g set has the effect of decreasing the synergistic effect of the back-bonding in both complexes. However, as the Fe complex presents higher character from ligand π-acceptor

orbitals, the stability brought by the back-bonding is decreased in this new state. The same goes for $[Co(CN)_6]^{3-}$, although to a lesser extent. As the stabilization brought by back-bonding in the cobalt is lower than in the Fe case, we believe the system is less perturbed by the ligand field excitation.

9) When you say a "smoothed version of the data…" please clarify what smoothing process has been applied and show what the unsmoothed data look like in the SI.

We thank the reviewer for the suggestion. We added in the subtitle of the figure a sentence mentioning we binned the data with a factor of 3. Furthermore, we added to the supporting information a plot of the data shown in Fig. 3d without data binning.

10) Overall, it might be that a rewritten version of the manuscript will make the arguments more clear, as I found the level of English to make it quite hard to follow (I will not comment specifically on the grammar, structure of paragraphs etc, but this should be considered much more carefully).

In the introduction, specify which complex has been studied, don't just say Fe(II) has been studied (I guess you're referring to Fe(bpy)3 etc).

The part in the introduction where it was stated "The Fe(II) presents…" was referring to Fe(II) as an instance within the hexacyanide class of complexes mentioned in the sentence prior. In the revised version of the text, "Fe(II)" was substituted by "$[Fe(CN)_6]^{-4}$" to make this clearer.

11) I think it should be called "the triplet state", not just "the triplet". This is should also be implemented in the title of the paper.

We thank the reviewer for this observation. We agree that it is a better way to refer to the triplet state and we have changed it in the title and throughout the revised manuscript.

12) Introduction: replace "spatial" with geometrical?

We have made the suggested change in the revised main text.

13) Co and Cobalt was used interchangeably in the manuscript. Please be consistent.

We thank the referee attention to this point. We revised the text and changed "Co" for "cobalt" except for when referring to the energy region between 775 and 805 eV which we call "Co $L_{3,2}$-edge" region of the electromagnetic spectrum for being a well known convention among our community.
